# Differential Conservation and Divergence of Fertility Genes *boule* and *dazl* in the Rainbow Trout

**DOI:** 10.1371/journal.pone.0015910

**Published:** 2011-01-06

**Authors:** Mingyou Li, Qian Shen, Hongyan Xu, Foong Mei Wong, Jianzhou Cui, Zhendong Li, Ni Hong, Li Wang, Haobin Zhao, Bo Ma, Yunhan Hong

**Affiliations:** 1 Department of Biological Sciences, National University of Singapore, Singapore, Singapore; 2 Heilongjiang Fisheries Research Institute, Ha'erbin City, China; 3 Yangtze River Fisheries Research Institute, Chinese Academy of Fishery Sciences, Jingzhou, China; University of Hong Kong, Hong Kong

## Abstract

**Background:**

The genes *boule* and *dazl* are members of the DAZ (*Deleted in Azoospermia*) family encoding RNA binding proteins essential for germ cell development. Although *dazl* exhibits bisexual expression in mitotic and meiotic germ cells in diverse animals, *boule* shows unisexual meiotic expression in invertebrates and mammals but a bisexual mitotic and meiotic expression in medaka. How *boule* and *dazl* have evolved different expression patterns in diverse organisms has remained unknown.

**Methodology and Principal Findings:**

Here we chose the fish rainbow trout (*Oncorhynchus mykiss*) as a second lower vertebrate model to investigate the expression of *boule* and *dazl*. By molecular cloning and sequence comparison, we identified cDNAs encoding the trout Boule and Dazl proteins, which have a conserved RNA-recognition motif and a maximal similarity to their homologs. By RT-PCR analysis, adult RNA expression of trout *boule* and *dazl* is restricted to the gonads of both sexes. By chromogenic and two-color fluorescence *in situ* hybridization, we revealed bisexual and germline-specific expression of *boule* and *dazl*. We found that *dazl* displays conserved expression throughout gametogenesis and concentrates in the Balbinani's body of early oocytes and the chromatoid body of sperm. Surprisingly, *boule* exhibits mitotic and meiotic expression in the male but meiosis-specific expression in the female.

**Conclusions:**

Our data underscores differential conservation and divergence of DAZ family genes during vertebrate evolution. We propose a model in which the diversity of *boule* expression in sex and stage specificity might have resulted from selective loss or gain of its expression in one sex and mitotic germ cells.

## Introduction

Gametogenesis, oogenesis in the ovary and spermatogenesis in the testis, proceeds in multiple processes including mitotic proliferation of germ stem cells and meiosis, as well as post-meiotic spermiogenesis in male. The DAZ (*Deleted in Azoospermia*) gene family represents one of the few lines of evidence for evolutionary conservation of these processes at the molecular level [1]. This family comprises *daz*, *dazl* and *boule*, which encode RNA-binding proteins characteristic of a conserved RNA recognition motif (RRM) and one or multiple repeats of the DAZ motif. The founder member of the DAZ family is the human Daz gene that forms a cluster on the Y chromosome [2], [3]. Daz has been thought of as being a male fertility factor, as deletion of the Daz cluster is causative for azoospermia and oligospermia. Besides the Y-chromosomal Daz, there is also a *Daz*-like gene on chromosome 3 (ENSG00000092345), termed *Dazla* [4], [5]. The Daz is limited to certain primates, whereas the autosomal *Dazl* homolog has been found in mouse [6]. The Dazla was proposed to be the ancestor of the Daz cluster, via its duplication and transposition to the Y chromosome during the evolution of primates [3]. The Dazl homolog has been found also in all major groups of non-mammalian vertebrates including chicken [7], *Xenopus* [8], axolotl [9], zebrafish [10], and medaka [11]. The third family member *boule* was identified in *Drosophila* as a gene essential for the germline development [12]. Similarly, the nematode *Caenorhabditis elegans* has a *boule* gene although it was initially called *daz*-1 [13]. The functional conservation between *dazl* and *boule* was demonstrated by the fact that transgenic expression of the *Xenopus dazl* was capable of rescuing the *boule* meiotic entry phenotype in *Drosophila* [8].

The DAZ family genes all show a conserved germ cell-specific expression [1]. They also share a conserved function in germline development from invertebrates to mammals, as demonstrated by the fact that the human *Dazla* can rescue the *Drosophila boule* mutant to some extent [14]. Since both *boule* and *dazl* exist in vertebrates, and only *boule* is present in invertebrates, *boule* has been hypothesized to be the ancestor of the DAZ gene family. According to this hypothesis, *boule* underwent gene duplication during vertebrate evolution, generating the autosomal *dazl*, which underwent further duplications, producing additional copies that were translocated to the Y-chromosome in primates [1].

On the other hand, the *DAZ* family genes exhibit a salient evolutionary variation in sex- and stage-specific expression. Specifically, *dazl* exhibits bisexual expression in diverse species, while the human *Daz* is specifically expressed in the male germline [1]. Intriguingly, *boule* expression is generally unisexual and shows considerable variation in opposite sexes across animal phyla: *boule* is expressed only in the male germline of *Drosophila* and mammals, whereas the nematode (*C. elegans*) *boule* (also called *daz-1*) is expressed in the female germline [15]. In all these organisms, *boule* expression is meiotic.

We are interested in the evolution of the DAZ family genes in fish as the most primitive group of vertebrates. Previously, we and others have identified *dazl* in zebrafish [16], gibel carp [17], medaka and stickleback [18], and *boule* in medaka and stickleback [18]. So far medaka is the only fish in which the RNA expression patterns of both *boule* and *dazl* have been analyzed in detail [18]. Interestingly, in this organism, not only *dazl* but also *boule* display bisexual expression in mitotic and meiotic germ cells [11], [18], raising a need for examining their expression in more organisms to better our understanding of the origin and evolution of DAZ family genes in vertebrates.

We are interested in the evolution of DAZ gene family in fish. Here we chose the rainbow trout (*Oncorhynchus mykiss*), which separated ∼200 millions years ago from the lineage leading to medaka [19], an evolutionary distance similar to that between human and platypus. The trout is a unique fish species for germ cell biology and biotechnology. In this organism, a *vasa* homolog has been identified as the first and only germ cell marker [20]. A transgenic trout line has been produced that expresses green fluorescent protein (GFP) from the trout *vasa* promoter [21] and allows for the isolation of primordial germ cells (PGCs) by fluorescence activated cell sorting from the larval gonad [22]. More importantly, transplantation of trout PGCs or testicular male germ cells into salmon embryos has led to successful surrogate reproduction [23], [24]. In this study, we identified *boule* and *dazl* in this organism and examined their RNA expression. We show that the trout *boule* and *dazl* have differential bisexual germ cell-specific expression and are markers for different stages of male and female gametogenesis.

## Results

### Gene identification

**Figure 1:**
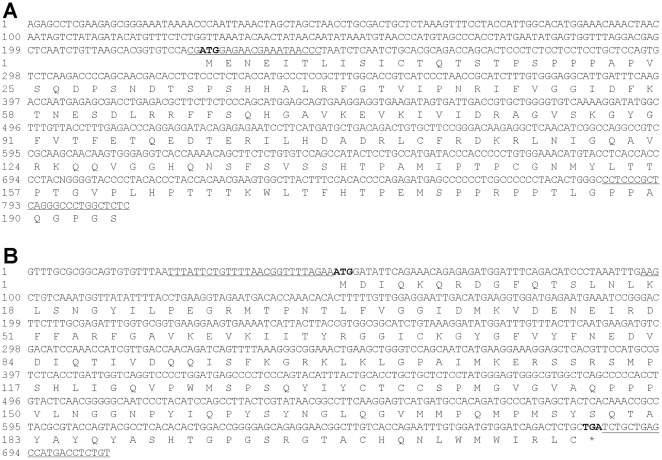
Nucleotide and amino acid sequences of the rainbow trout *boule* and *dazl*. (A) *boule*. (B) *dazl*. Start and stop codons are in bold. Sequences underlined are primer sequenced used for cDNA cloning and RT-PCR analysis, which also flank the regions used for the synthesis of RNA probes for in situ hybridization.

To identify trout *boule* and *dazl*, we exploited molecular cloning and bioinformatics approach. BLAST search by using the medaka sequences against available libraries of trout expressed sequence tags led to the identification of two cDNA sequences as the putative trout *boule* (HQ696915) and *dazl* (HQ696914). Both cDNAs were PCR-cloned by using primers complementary to the termini and sequenced. The *boule* cDNA is 808 nt in length, contains a 216-nt 5′-untranslated region (UTR) and a partial open reading frame (ORF) of 585 nt for 195 amino acid residues [25] (Figure 1). The predicted trout Boule displays a maximal identity of 77% and 54% to the medaka and mammalian Boule proteins. The *dazl* cDNA is 706 nt, contains a 48-nt 5′-UTR and a 651-nt full open reading frame (ORF) for 212 aa (Figure 1B). The predicted trout Dazl is maximally 77% and 54% identical to the medaka and mammalian Dazl proteins.

**Figure 2:**
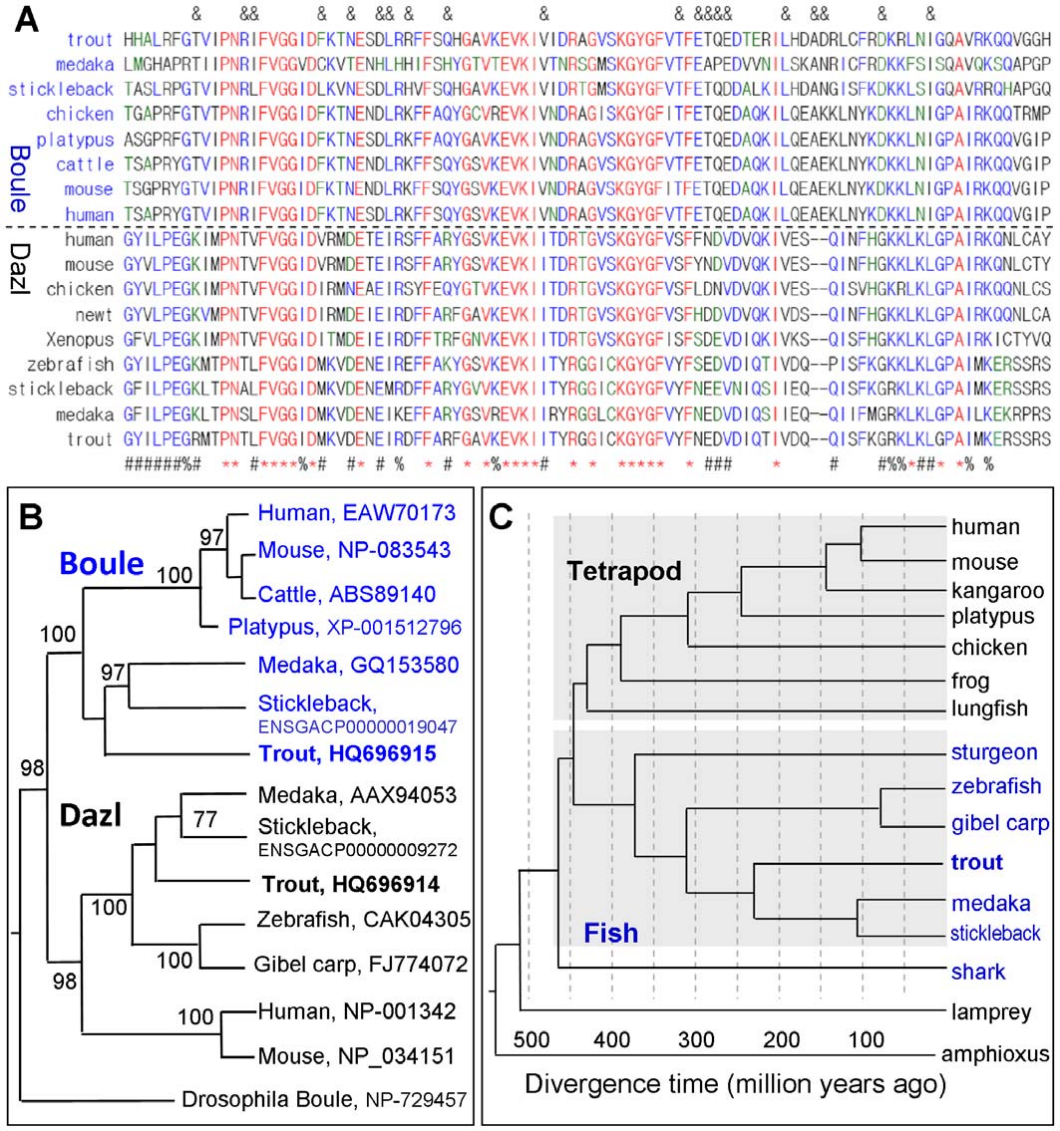
Comparisons of Boule and Dazl proteins between rainbow trout and other organisms. (A) Multiple sequence alignment of the RRM. Boule and Dazl proteins share 16 invariant residues (asterisks) and 13 conserved positions (%). There are 21 invariant or conserved residues characteristic of Boule (&) and Dazl (#) proteins each. (B) Phylogenetic tree of DAZ family proteins. The branching between Boule and Dazl coincides with the branching between the fish and tetrapod lineages, and molecular trees on the basis of Boule and Dazl sequences are in accordance with organism relationships, indicating that generation of *boule* and *dazl* took place in early vertebrate evolution. Followed species are gene accession numbers. (C) Organism phylogeny and branching times of major fish groups and tetrapod vertebrates. Fish timing is according to [19].

The conserved positions between the trout Boule or Dazl and their respective homologs do not evenly distribute along entire molecules but instead reside within the RRM. Therefore, we focused our comparison on a multiple sequence alignment of the RRM only. This revealed that Boule differs from Dazl in 20 invariant and/or conserved positions, besides 27 invariant and/or conserved positions common to both Boule and Dazl (Figure 2A). These conserved positions are the same as those previously identified [18]. On a phylogenetic tree, Boule proteins are clustered together, whereas all Dazl forms a separate clade (Figure 2B). Interestingly, the Boule-Dazl branching coincides with separation between fish and tetrapod lineages (Figure 2C). This, together with the fact that fish Boule is more similar to mammalian Boule than to fish Dazl, strongly supports the early divergence of vertebrate *boule* and *dazl* before fish-tetrapod separation.

### Bisexual RNA expression

**Figure 3:**
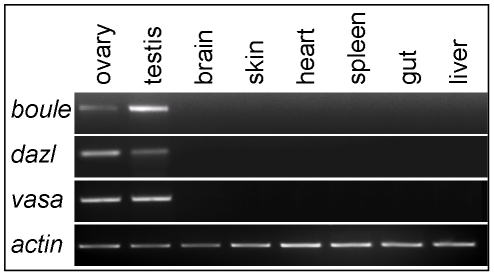
RT-PCR analysis of trout *boule* and *dazl* RNA expression. The RNA is abundant in the female gonad ovary and male gonad testis but absent in all somatic tissues examined.

In many species so far examined except cattle [26], *dazl* has bisexual germline expression. However, *boule* has considerable diversity, ranging from unisexual expression in male fly [12], male mammals [1], but female worm [15] and bisexual expression in medaka [18]. By RT-PCR, the transcripts of trout *boule* and *dazl* were found to be absent in somatic tissues but high in adult gonads of both sexes (Figure 3), suggesting that adult *boule* and *dazl* expression may be restricted to germ cells.

### Germ cell-specific expression during oogenesis

**Figure 4:**
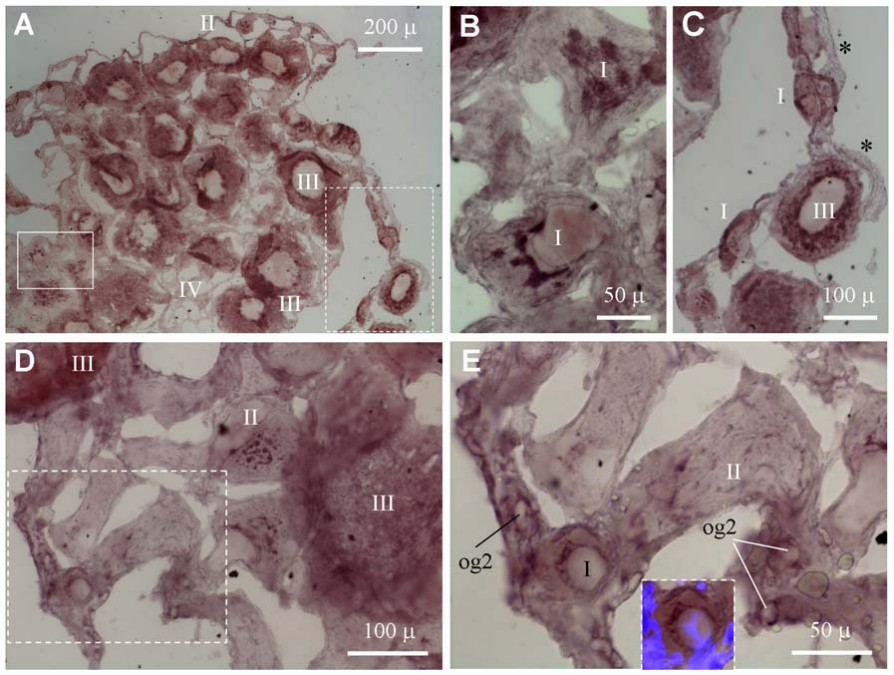
Expression of *boule* RNA during oogenesis. Adult ovarian cryosections were hybridized to antisense RNA probes and the signals were visualized by chromogenic staining. (A) Lower magnification view showing different stages of oocytes (I to IV). (B) Larger magnification of the area framed by solid line in (A), highlighting speckles of *boule* RNA in stage-II oocytes. (C) Larger magnification of the area framed by broken line in (A), highlighting the absence of *boule* RNA speckles in oocytes at stage I and III, and the lack of *boule* signal in somatic cells (asterisks). (D) Oogonia and early oocytes. (E) Larger magnification of the framed area in (D), highlighting differentiating oogonia (og2) and early oocytes at stage I with residual nuclear staining by DAPI (inset).

We performed *in situ* hybridization (ISH) to examine the *boule* and *dazl* RNA expression during gametogenesis and compared with *vasa*, the well-studied germ cell marker. The adult trout ovary comprises a small number of oogonia and oocytes and oogenesis proceeds in stages I to IV. By chromogenic ISH, the *boule* RNA was found to be restricted to germ cells but barely detectable in surrounding somatic cells (Figure 4A–C). Notably, it is hardly detectable in undifferentiated oogonia and becomes detectable in differentiating oogonia seemingly entering into meiosis, increases in meiotic female germ cells, namely stage I-III oocytes (Figure 4D and E). In addition, the *boule* RNA displays a dynamic subcellular distribution relative to stages of oogenesis: It disperses evenly in small oocytes at stage I, concentrates in several patches in growing oocytes at stage II, and re-disperses again in oocytes from stage III onwards (Figure 4A–C).

**Figure 5:**
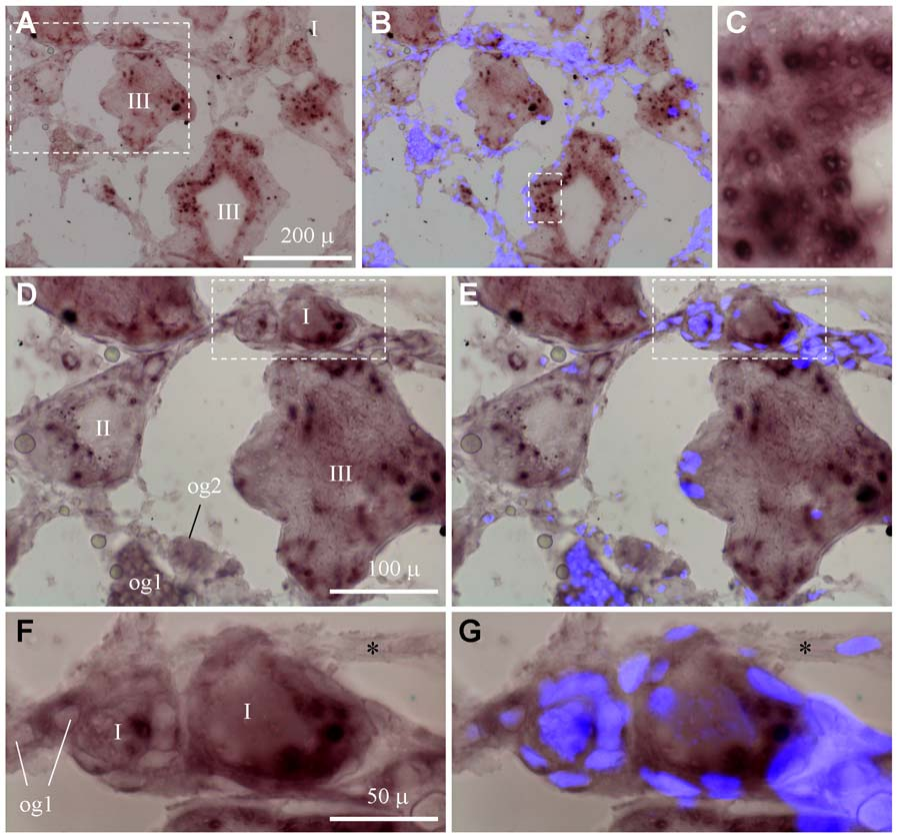
Expression of *dazl* RNA during oogenesis. (A and B) Lower magnification view showing different stages of oocytes (I to III) and dot-like structures of *dazl* RNA. (C) Larger magnification of the framed area in (B), highlighting hollow-like structure of *dazl* RNA. (D and E) Larger magnification of the framed area in (A), highlighting a cluster of undifferentiated oogonia (og1) and larger-sized, differentiating oogonia (og2). (F and G) Larger magnification of the framed area in (D and E), highlighting oogonia (og1) and early oocytes at stage I with residual nuclear staining by DAPI, and the absence of *dazl* signal in somatic cells (asterisk).

At lower magnifications, the *dazl* signal was found to concentrate in speckles at a low magnification (Figure 5A and B). These speckles appear to be hollow-like structures at a higher magnification (Figure 5C). The *dazl* RNA is also restricted to germ cells and barely detectable in surrounding somatic cells (Figure 5D–G). Unlike *boule*, the *dazl* RNA persists throughout oogenesis, with its signal being clearly visible in undifferentiated oogonia (Figure 5D–G). The *dazl* RNA also displays a dynamic subcellular distribution different from *boule*. In oogonia, *dazl* RNA distributes evenly without forming speckles (Figure 5F and G).

### Differential expression during oogenesis

**Figure 6:**
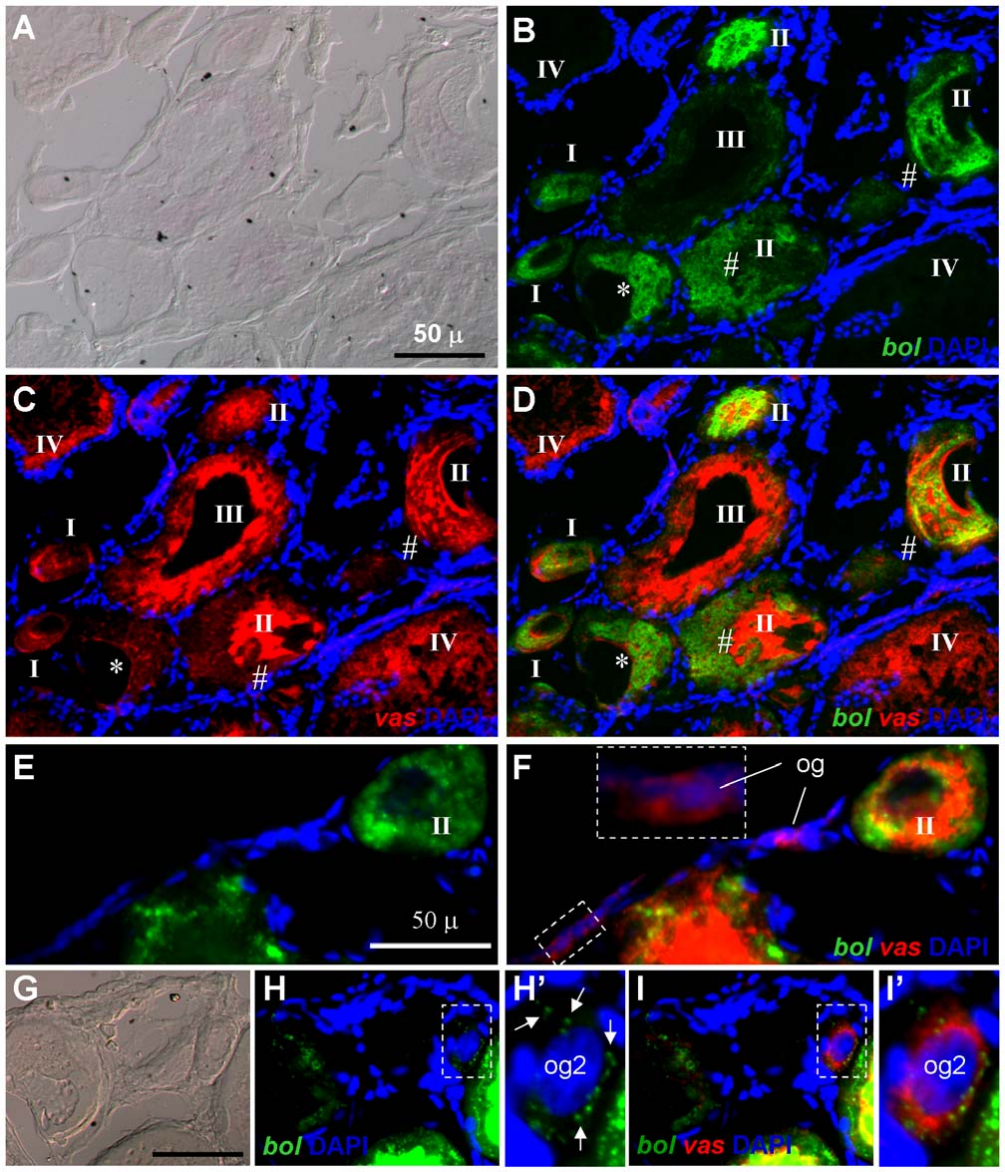
Expression of *boule* and *vasa* transcripts in trout oogenesis. Adult ovarian cryosections were hybridized to antisense RNA probes and the signals were visualized by fluorescence staining. Nuclei were stained blue by DAPI. (A–D) Stages I–IV of oocytes. Oocytes showing co-distribution (*) and alternative distribution (#) are indicated. (E and F) Oogonia. (G–I') Differentiating oogonium entering into meiosis, highlighting the onset of *boule* RNA expression at this stage. (H' and I') Larger magnifications of framed areas in (H and I). This oogonium retains nuclear staining by DAPI and contains a few speckles of *boule* RNA (arrows). Scale bars, 50 µm.

After verifying the specificity of both *boule* and *dazl* probes by chromogenic ISH, we wanted to precisely compare the RNA expression patterns of *boule* and *dazl* together with *vasa* at different stages of oogenesis. The *vasa* expression has been demonstrated to germline-specific [20], [21]. To this, we developed in the trout a two-color fluorescence *in situ* hybridization (FISH) procedure. We first compared *boule* and *vasa* by FISH (Figure 6). The *vasa* RNA persists throughout oogenesis. The *vasa* signal is relatively weak in stage-I oocytes and become most abundant in oocytes at stages II and III (Figure 6A–D). At higher magnification, the *vasa* signal is easily detectable also in undifferentiated oogonia (Figure 6E and F) and differentiating oogonia seemingly entering into the meiosis (Figure 6G–I'). The *boule* signal is visible in early oocytes at stage I, peaks at stage II, declines at stage III and dramatically decreases at stage IV (Figure 6A–D). A closer inspection at a higher magnification revealed that the onset of detectable *boule* RNA expression occurred in differentiating oogonia (Figure 6G and H), in which the *boule* signal is seen as small particles surrounding the nucleus (Figure 6H' and I'). These differentiating oogonia are characteristic of a larger size (∼30 µm in diameter compared to ∼20 µm for undifferentiated oogonia) and strong DAPI staining. DAPI does not stain oocyte nuclei. A larger size and remaining DAPI staining suggest that these are differentiating oogonia entering into meiosis. Generally, the *vasa* signal is stronger than the *boule* signal except for stage I, where *boule* is stronger than *vasa*. Notably, *boule* and *vasa* RNA display independent subcellular distribution. This is most evident in stage-II oocytes, where the RNAs of both genes may exhibit co-distribution or alternative distribution (Figure 6).

**Figure 7:**
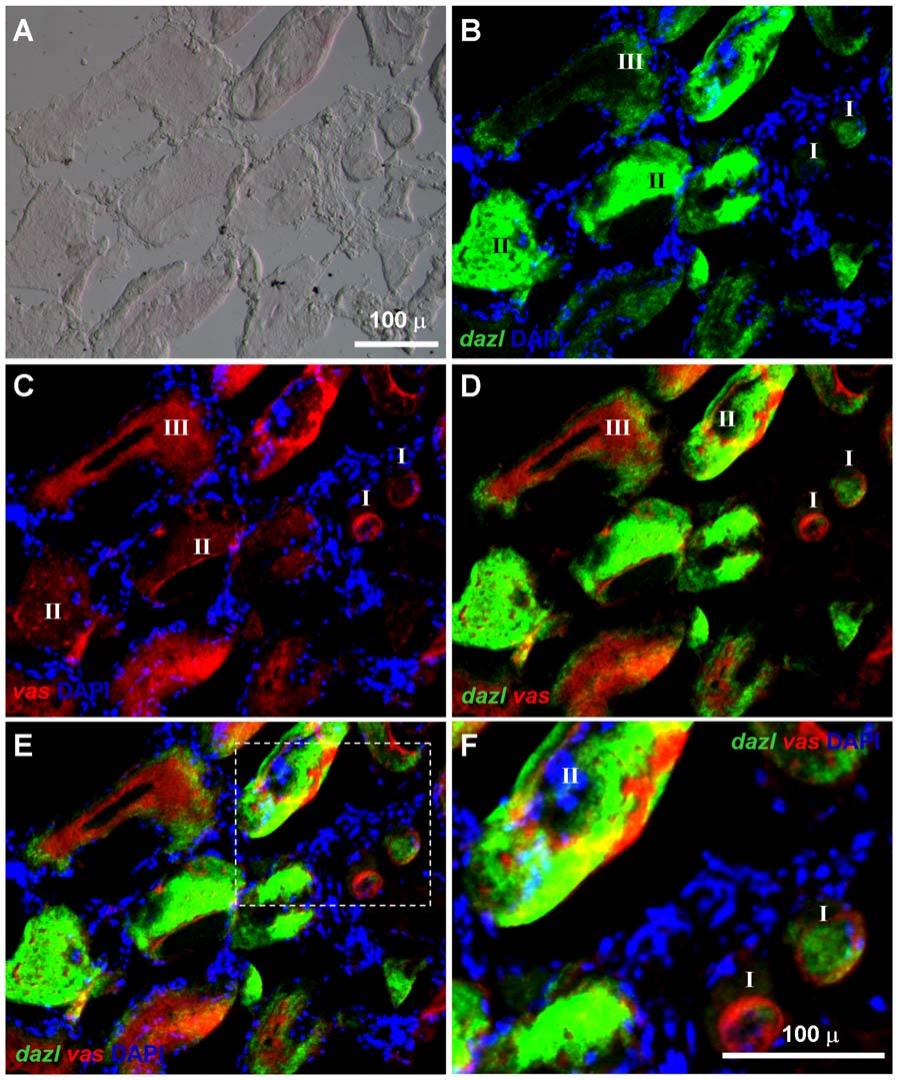
Expression of trout *dazl* and *vasa* RNA in trout oogenesis. After hybridization with antisense trout *dazl* and *vasa* RNA probes, the signals were visualized by fluorescence staining. Nuclei were stained blue by DAPI. (A–D) Bright-field, green fluorescent, red fluorescent optics and merge, respectively, showing independent subcellular distribution of *dazl* and *vasa* RNAs. Scale bars, 100 µm.

**Figure 8:**
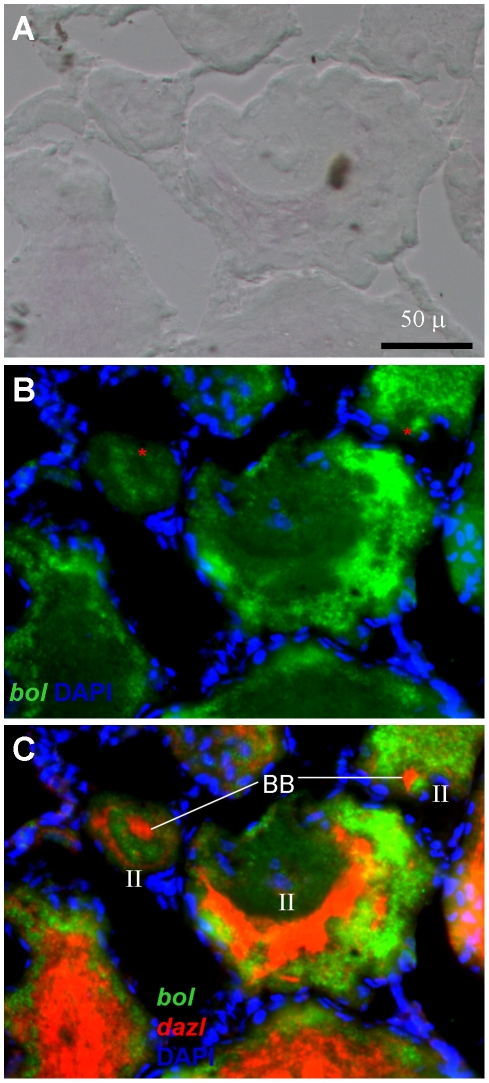
Expression of *boule* and *dazl* transcripts in oogenesis. (A) Bright field. (B) *boule* signal. (C) *boule* and *dazl* signals. Notably, the *dazl* but not *boule* RNA is seen to be concentrated in the Balbiani's body (BB).

We then examined the *dazl* and *vasa* by FISH (Figure 7). This revealed a similar expression pattern and also independent subcellular distribution for both genes, with some oocytes predominantly showing the *dazl* or *vasa* signal at the focus of section/observation (Figure 7A–D). We finally analyzed *boule* and *dazl* by FISH (Figure 8). We observed again independent subcellular distribution, predominantly mosaic distribution (Figure 8). Another salient observation was made in stage-II oocytes. In these oocytes, *dazl* concentrates in a cytoplasmic structure, the so-called Balbiani's body (BB), where *boule* is essentially absent (Figure 8). BB is a spherical membrane-less structure, forming in contact with the oocyte nucleus in early oogenesis [27], [28], [29] and containing mitochondria, endoplasmic reticulum and *dazl* mRNA during early oogenesis in *Xenopus* [27], zebrafish [16] and medaka [18]. Therefore, differential concentration in the BB is a conserved divergence between *boule* and *dazl* RNAs in trout and other organisms.

### Differential expression during spermatogenesis

**Figure 9:**
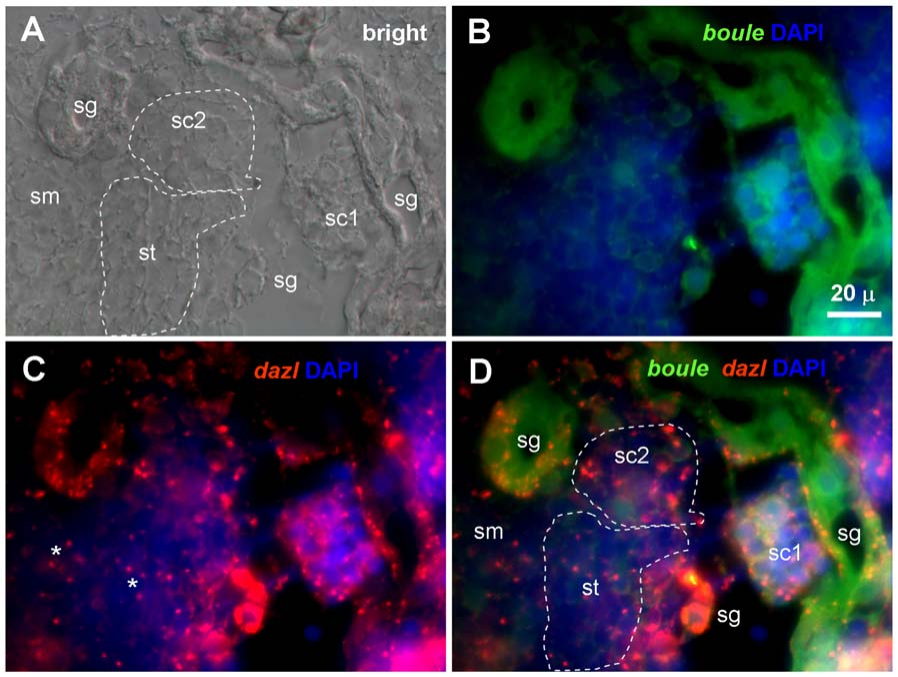
Expression of *boule* and *dazl* transcripts in trout spermatogenesis. Adult testicular cryosections were hybridized to antisense RNA probes and the signals were visualized by fluorescence staining. Nuclei were stained blue by DAPI. (A) Bright field. (B) *boule* signal. (C) *dazl* signal. (D) Merge. Different stages of spermatogenesis are indicated: sg, spermatogonia; sc1, primary spermatocytes; sc2, secondary spermatocytes; st, spermatids; sm, sperm. The *boule* RNA forms the diffuse signal, whereas the *dazl* RNA forms speckles in spermatogonia and spermatocytes, and condenses in the chromatoid body (asterisks) of spermatids and sperm. Scale bars, 20 µm.

We furthered our experiments to examine the expression of *boule* and *dazl* by FISH during spermatogenesis in the adult testis. The expression of *boule* and *dazl* was restricted to male germ cells but absent in surrounding somatic cells (Figure 9). Importantly, both the *boule* and *dazl* signal peak in spermatogonia, in sharp contrast to female expression of *boule*, which is meiosis-specific but absent in oogonia of the mitotic phase. Another difference was seen in postmeiotic spermatids, where the *boule* signal was hardly detectable, and the *dazl* signal persisted.

**Figure 10:**
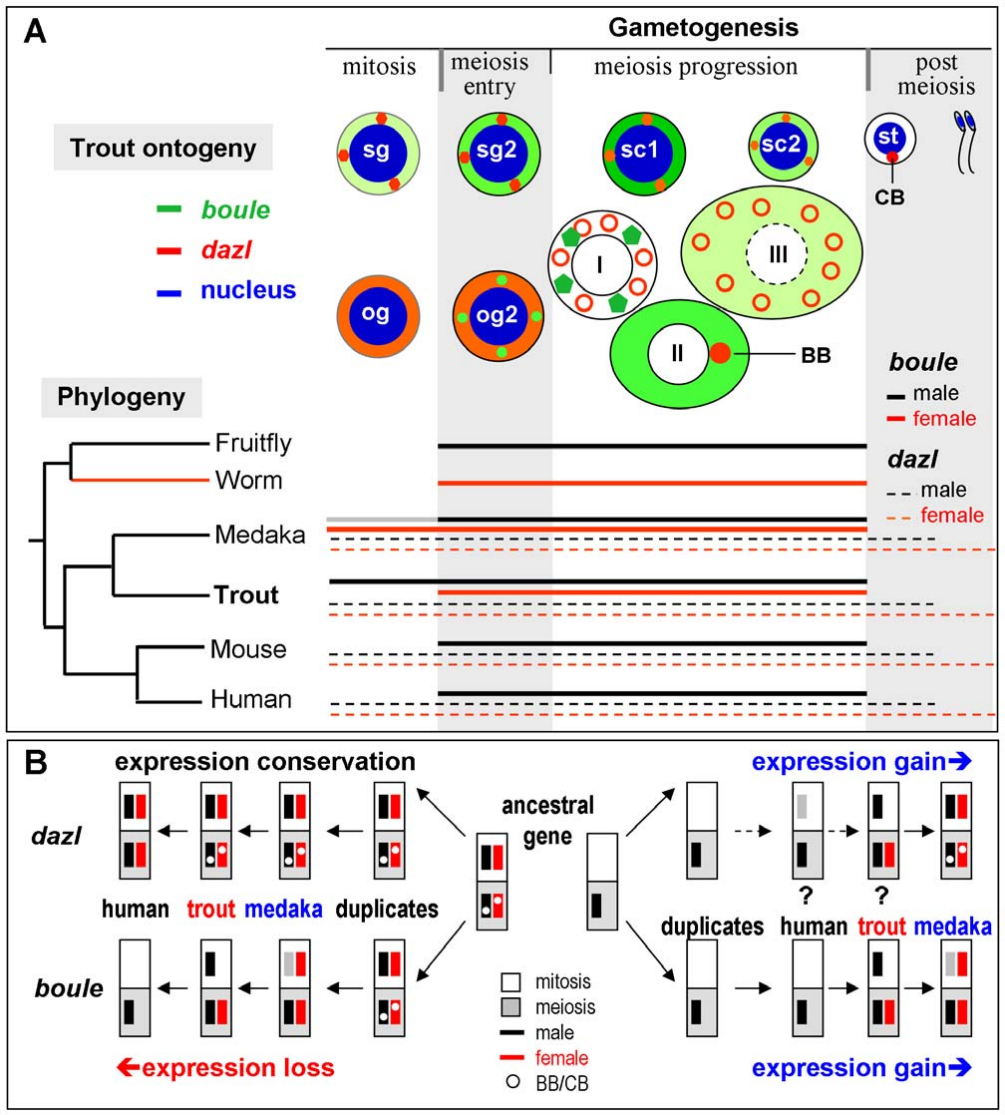
Evolutionary divergence of *boule* expression. (A) Left, Phylogeny. The ancient member *boule* exists in all metazoans. Sex specificity of expression is indicated different colors (male, black; female, red; color intensity, relative expression level). Right, gametogenic expression. Expression pattern is indicated by extent of horizontal lines. Major stages of gametogenesis are diagramed as a timeline for *boule* gene expression. Unisexual meiotic expression of *boule* occurs in male fly, mouse and human (most abundantly in primary spermatocytes) and in female worm. Our previous work and this study demonstrate that bisexual *boule* expression occurs in both fish species. Data obtained in this study from trout clearly demonstrate that *boule* expression in the ovary is restricted to oogenic meiosis, in contrast to both premeiotic and meiotic expression in medaka. Notably, the male mitotic expression of *boule* is weaker than its meiotic expression. (B) Alternative models of evolution. In the loss of expression model, the ancestral gene and its initial duplicates have bisexual expression throughout gametogenesis: One duplicate has conserved the original expression pattern and become *dazl*, whereas the other duplicate has undergone selective loss of expression and become *boule* that ultimately displays male meiotic expression in mammals [1]. In the gain of expression model, a reversed situation applies. The situations we found in medaka [11], [18] and trout (this study) appear to represent two intermediate steps of *boule* evolution and supports either model. Highly conserved bisexual *dazl* expression in mitotic and meiotic germ cells favors the gain of expression model, whereas unisexual meiotic expression of *boule* in mammals and more importantly, in invertebrates as the only family member seemingly supports the gain of expression model. Question marks denote hypothetical intermediate steps in the gain of expression model.

Differential subcellular distribution of *boule* and *dazl* is also evident in the testis. In all these spermatogenic cells, the *boule* distributed evenly in the cytoplasm, whereas the *dazl* RNA concentrated in speckles in spermatogonia and spermatocytes (Figure 10). Strikingly, in post-meiotic spermatids, the *dazl* RNA localizes in a structure called the chromatoid body (CB) (Figure 9C and D). CB is a unique membrane-less cytoplasmic structure in spermatids and sperm, which contains germ plasm RNA and protein components such as *vasa* [29].

## Discussion

In the present study, we have identified the trout *boule* and *dazl* and analyzed their expression in detail during oogenesis and spermatogenesis. Several lines of evidence, including phylogenetic sequence comparisons, protein structure and expression patterns, support the notion that the trout *boule* and *dazl* are orthologous to the known *boule* and *dazl*, respectively. With the identification of mammalian *boule*, the DAZ gene family was proposed to have evolved from *boule* by gene duplication and/or translocation [1]. Our previous work on medaka *boule* and *dazl* provided a first direct evidence for ancient gene duplication during early vertebrate evolution, prior to the separation between fish and tetrapod lineages approximately 450 million years ago (Figure 10A). The identification of trout *boule* and *dazl* and their expression analyses in this study underscore the notion that *boule* and *dazl* coexist widely in vertebrates, and that *boule* and *dazl* become paralogs of each other since their initial duplication event in a common ancestor.

In this study, we have developed a two-color FISH procedure for the simultaneous analysis of multiple RNA molecules at a high sensitivity. By using this procedure we revealed several important observations on the differential expression of trout *boule* and *dazl* during oogenesis and spermatogenesis. First, both genes exhibit a bisexual germline-specific expression, similar to the *vasa*, the best studied germ cell marker in diverse organisms including the zebrafish [30], [31], medaka [32], gibel carp [33] and trout [20], [21]. Therefore, the germline-specific expression of the DAZ family genes has generally been conserved in invertebrates and vertebrates.

Second, the trout *dazl* shows not only expression in the mitotic and meiotic phases of both female and male gametogenesis but also stage-specific subcellular localization into the Balbiani's body and chromatoid body, cytoplasmic structures that are thought as being stage-specific germ plasm in the oocyte and sperm [18], [29]. This expression pattern and subcellular localization conforms to those reported for its homolog in three other fish species, namely medaka [18], gibel carp [17] and zebrafish [30], [31], and several representatives of tetrapod vertebrates including *Xenopus* [8], chicken [34] and mammals [1]. Thus, the bisexual mitotic and meiotic germline expression and subcellular localization we revealed for the medaka and trout *dazl* gene must represent the characteristics of the vertebrate *dazl* gene prototype, which have been highly conserved during evolution of vertebrates from fish to mammals.

Finally and most importantly, we have made unusual observations on the trout *boule* expression. In diverse organisms, *boule* is best known for its unisexual meiotic expression and requirement in invertebrates. In vertebrates, *boule* shows considerable variations, ranging from male-only meiotic expression in mammals to bisexual expression in embryonic germ cells and adult germ cells at the mitotic and meiotic phases of medaka oogenesis and spermatogenesis. In this study, *boule* expression is bisexual in trout similar to medaka. In the testis, the trout *boule* also resembles its medaka homolog in weak expression in spermatogonia of the mitotic phase, strong expression in spermatocytes of the meiotic phase and rapid disappearance in spermatids/sperm of the meiotic phase. These suggest that *boule* has evolved meiosis-preferential expression in male germ cells of both trout and medaka. In the trout ovary, however, *boule* expression is absent in undifferentiated oogonia even by sensitive FISH, in contrast to medaka *boule* that shows easily detectable expression together with *dazl* throughout oogenesis including oogonia [18]. Interestingly, trout *boule* commences expression in differentiating oogonia seemingly entering into meiosis and dramatically increases in the early meiotic phase as seen in oocytes at stages I and II. It is these early oocytes that express an even higher level of RNA for *boule* than *dazl*. Moreover, meiosis progression into stages-III oocytes accompanies a decrease in *boule* expression. In addition, the trout *boule* resembles its medaka homolog in the absence of localization into the Balbinani's body and chromatoid body. These data demonstrate that female *boule* expression in trout is meiotic and occurs predominantly in the early meiotic phase. Therefore, the present work provides two new germ cells markers to identify different stages of gametogenesis in trout for germ cell biology and reproductive technology.

The two-color procedure also led to the observation that the trout *boule*, *dazl* and/or *vasa* exhibit independent subcellular distribution instead of colocalization during gametogenesis, which is most evident in early oocyte development. Throughout spermatogenesis, *boule* diffuses and *dazl* forms speckles. During oogenesis, *boule* also diffuses and concentrates in speckles in stage-II oocytes, whereas *dazl* localizes in many speckles of hollow-like structures again in stage-II oocytes. This observation in trout further adds the diversity to the expression of DAZ family genes.

Our detailed comparative expression analyses of the DAZ family genes in medaka [11], [18] and trout (this study) as two representatives of primitive vertebrates shed important lights on the evolution of this family in vertebrates (Figure 10A). The meiotic-specific female expression plus meiosis-preferential male expression of trout *boule*, and meiosis-preferential bisexual expression of medaka *boule*, together with the highly conserved bisexual expression of *dazl* throughout gametogenesis in diverse species, point to differential conservation and divergence of the DAZ family genes in vertebrates. We propose two alternative models for the evolution of differential *boule* and *dazl* expression in diverse species (Figure.10B). In a loss of expression model, the common ancestral gene had bisexual mitotic and meiotic expression, and the ancient gene duplication generated *boule* and *dazl*, which were initially identical in bisexual expression but subsequently underwent different ways of evolution: *dazl* shows conservation, whereas *boule* exhibits divergence towards unisexual meiotic expression by loss of mitotic expression, loss of female expression and loss of subcellular localization, ultimately leading to meiotic male *boule* expression as seen in mammals. According to this loss of expression model, *boule* has evolved by selective loss of expression, whereas *dazl* has conserved the original expression. A gain of expression model would also apply to the DAZ family evolution: gain of mitotic expression, gain of female expression and gain of subcellular localization, resulting in *dazl* as seen diverse species. The situations we found in medaka and trout appears to represent two intermediate steps of *boule* evolution and supports either model. Highly conserved bisexual *dazl* expression in mitotic and meiotic germ cells favors the gain of expression model, whereas unisexual meiotic expression of *boule* in invertebrates as the only family member seemingly supports the gain of expression model. Future examination of DAZ family genes in more primitive vertebrates and in species closely related to vertebrates will offer valuable information to distinguish the two models.

It has been documented that the expression of the DAZ family genes well correlates with their specific roles in different stages of germline development. For example, the *Drosophila boule* is expressed in meiotic male germ cells and required for the meiotic progression in spermatogenesis but not oogenesis, as mutant male flies have infertility with their male germ cells arrested at the G2/M transition in meiosis I [12]. The nematode *boule* homolog is expressed in meiotic female germ cells, and its mutations completely abolish fertility in hermaphrodites due to arrest at meiotic prophase in oogenesis [15]. Targeted disruption of mouse Dazla completely abolishes gamete production in both sexes, with female germ cells being arrested at the prophase of meiosis I and male germ cells being affected at the proliferating stage [35]. It has recently reported in cell cultures that the human Dazl functions in primordial germ cell formation, whereas Daz and Boule promote later stages of meiosis and development of haploid gametes [36], in accordance with their stage-specific expression [1]. Comparative expression analyses of the DAZ family genes in lower and higher vertebrates will provide useful information on the evolution and function of molecular mechanisms underlying gametogenesis and fertility.

## Materials and Methods

### Fish and Chemicals

This study was carried out in strict accordance with the recommendations in the Guide for the Care and Use of Laboratory Animals of the National Advisory Committee for Laboratory Animal Research in Singapore and approved by this committee (Permit Number: 27/09). Rainbow trout females were purchased from a fish farmer in Wϋrzburg, Germany, and adult testis samples were kindly provided by Dr. Goro Yoshizaki (Japan) and the Heilongjiang Fisheries Research Institute (Ha'erbin, China). Chemicals and enzymes were from Sigma and Promega, respectively, unless otherwise indicated.

### Isolation of cDNA sequence

Total RNA was isolated from adult tissues by using the Trizol Reagent (Invitrogen). To eliminate genomic DNA contamination, RNA samples were treated with RNase-free DNase (Promega). cDNA was synthesized by using MMLV reverse transcriptase (Invitrogen) as described [11]. PCR was performed by using two primers each for *boule* (bolf, CGATGGAGAACGAAATAACCC; bolr, GAGAGCCAGGGCCCTGAGCGGGAGG), *dazl* (dazf, ATTTATTCTGTTTTAACGGTTTTAGAA; dazr, ACAGAGGTCATGGCTCAGCAGA) and *vasa* (vasf, GGGACCTCATGGCCTGTGCC; vasr, ATCACTCCCATTCGTCGTCG; accession number, AB032566.1). As a control, β-actin was amplified from the same set of cDNA samples by using two primers TA1, TTCAACAGCCCTGCCATGTAC; TA2 (CCTCCAATCCAGACAGAGTATT) according to the trout actin cDNA sequence (accession number, AF157514.1). A PCR reaction in a 20 µl volume contained a cDNA aliquot equivalent to 20 ng of total RNA. PCR was for 35 cycles of 20 s at 94°C, 20 s at 58°C and 60 s at 72°C. The PCR products were separated on 1.2% agarose gels and documented with a bioimaging system (Synoptics).

### Sequence Analysis

The PCR products of the 808-nt *boule* cDNA, 706-nt *dazl* cDNA and 1.2-kb *vasa* cDNA were cloned into pGEM-T vector, resulting in pOMbol, pOMdazl and pOMvasa. The inserts were sequenced in both directions on the Applied Biosystems 3130xl (Applied Biosystems, MA). BLAST searches were run against public databases by using BLASTN for nucleotide sequences and BLASTP for protein sequences. Multiple sequence alignment was conducted by using the Vector NTI suite 8. Phylogenetic trees were constructed by using the DNAMAN package.

### Sections

The trout testis and ovary were fixed overnight or longer at 4% paraformaldehyde (PFA) in phosphate-buffered saline (PBS) at 4°C. They were dehydrated by passing through an increasing series of ethanol (70%, 80%, 90% and 100%) for 1 hour each. After an additional treatment in 100% ethanol, the samples were treated for 30 min each in a mix of Histoclear:ethanol (1∶1) and 100% Histoclear. After 30-min incubation in paraffin at 60°C, the samples were treated overnight in paraffin at 60°C and embedded in paraffin. The testis was cut at 5 µm and ovary at 8 µm on the Leica RM2135 Microtomes (Leica, Germany). Slides of sections were dewaxed by immersion in xylene with three changes, each for 10 min. After rehydration through a descending ethanol series (100%, 90%, 70%, 50% and 30%), the slides were subjected to treatments two times with PBS containing 0.1% Tween-20 (PBST) for 5 min each, once with 0.1N HCl for 5 min and three washes in PBST for 5 min each. The samples were treated with proteinase K (10 µg/ml) for 30 min at 37°C, washed three times in PBST for 5 min each, and refixed in 4% for 15 min at room temperature. After three PBST rinses, the samples were subjected to in situ hybridization.

### Chromogenic i*n situ* hybridization

Chromogenic ISH by using BCIP/NBT as substrates was performed as described previously with minor modifications [33]. Briefly, pOMbol, pOMdazl and pOMvasa were linearized with Xho I and Sac II and used for the synthesis of sense and anti-sense RNA probes from SP6 or T7 promoter by using the digoxigenin (DIG) Labeling Kit (Roche) for *dazl* and *vasa*, or the FITC RNA Labeling Kit (Roche) for *boule*. The RNA probes were treated with RNase-free TURBO DNase (Ambion) and purified by using the LiCl method according to the supplier's instruction (cat #AM1340, Ambion). DIG probe and FITC probes were denatured incubation at 90°C for 5 min followed by rapid cooling on ice. Chromogenic ISH was performed as described previously [33]. Briefly, the probe was added at ∼1 µg/ml to the hybridization buffer (50% formamide, 5× SSC, 500 µg/ml yeast tRNA, 0.1% Tween-20 and 50 µg/ml heparin). The samples on each slide were covered with 100 µl of the hybridization buffer containing a probe and wrapped in parafilm. The slides were put into a Petri dish layered at bottom a Whatman paper presoaked with 2× SSC containing 0.1% Tween-20 (SSCT) and 50% formamide. The dish was sealed with parafilm and incubated overnight at 65°C. The slides were washed two times in 2× SSCT-50% formamide for 30 min each, two times in 2× SSCT and two times in 0.2× SSCT for 30 min each. After incubation in blocking buffer (PBST containing 2% blocking reagent and 10% goat serum) for 1 hour at room temperature, the samples were incubated with either the alkaline phosphatase (AP) conjugated anti-DIG-antibody or AP-conjugated anti-FITC-antibody (Sigma) at 1∶2000 in the blocking buffer for 2 hours at room temperature. Following six PBST washes, and preincubation 2 times in the pre-staining buffer (NTMT; 100 mM NaCl, 50 mM MgCl_2_, 100 mM Tris-Cl, pH 9.5, 0.1% Tween-20) for 15 min each, the sample were incubated in the staining buffer (0.1 mg/ml NBT/BCIP in NTMT) in darkness at room temperature for 30–60 min or at 4°C overnight. Color development was microscopically monitored at regular intervals. Nuclear staining was done by using 4′–6 Diamidino-2-phenylindole (DAPI; 1 µg/ml) and embedded in the Gold Antifade reagent (Invitrogen) for microscopy.

### Fluorescence *in situ* hybridization

Two-color fluorescence in situ hybridization (FISH) was performed by using the tyramide signal amplification (TSA™) Plus Fluorescence Systems according to the manufacturer's instruction (NEL756, PerkinElmer Life Science) [18]. Briefly, after hybridization and blocking as described above, the samples were incubated with the horseradish peroxidase (POD)-conjugated anti-FITC-antibody (Sigma) at a 1∶2000 dilution for 2 hours at room temperature to detect the FITC-labeled probes. Following six PBST washes the samples were incubated for 30 minutes in the TSA-Fluorecein at a 1∶100 dilution in the TSA Amplification Buffer (0.004% H_2_O_2_ in 0.1 M borate buffer, pH 8). The samples were then subjected to detection of the DIG-labeled probe: They were treated for 1 hour in blocking buffer with 1% H_2_O_2_ and incubated for two hours with the POD-conjugated anti-DIG antibody (Sigma) at a 1∶2000 dilution, followed by incubation in TSA-Cy3 for 30 min. The samples were finally stained for nuclei by using DAPI and embedded in the Gold Antifade reagent (Invitrogen) for microscopy. In this FISH procedure, two differently labeled antisense RNA probes are co-incubated with a sample in the hybridization step and subjected to sequential color development into green and red fluorescence, and nuclei are stained with DAPI for blue fluorescence. This procedure features multiple combinations between brightfield optics and three colors of fluorescent optics, allowing for precise comparisons of the relative signal intensity and distribution of different molecules. FISH in medaka provides detection sensitivity ∼100 times that of the chromogenic ISH [18], [37].

### Microscopy and Photography

Observation and photography on Leica MZFIII stereo microscope, Zeiss Axiovertinvert and Axiovert upright microscopes with a Zeiss AxioCam M5Rc digital camera (Zeiss Corp) were as described previously [11].

## References

[1] Xu EY, Moore FL, Pera RA (2001). A gene family required for human germ cell development evolved from an ancient meiotic gene conserved in metazoans.. Proc Natl Acad Sci U S A.

[2] Reijo R, Lee TY, Salo P, Alagappan R, Brown LG (1995). Diverse spermatogenic defects in humans caused by Y chromosome deletions encompassing a novel RNA-binding protein gene.. Nat Genet.

[3] Saxena R, Brown LG, Hawkins T, Alagappan RK, Skaletsky H (1996). The DAZ gene cluster on the human Y chromosome arose from an autosomal gene that was transposed, repeatedly amplified and pruned.. Nat Genet.

[4] Reijo R, Seligman J, Dinulos MB, Jaffe T, Brown LG (1996). Mouse autosomal homolog of DAZ, a candidate male sterility gene in humans, is expressed in male germ cells before and after puberty.. Genomics.

[5] Seboun E, Barbaux S, Bourgeron T, Nishi S, Agulnik A (1997). Gene sequence, localization, and evolutionary conservation of DAZLA, a candidate male sterility gene.. Genomics.

[6] Cooke HJ, Lee M, Kerr S, Ruggiu M (1996). A murine homologue of the human DAZ gene is autosomal and expressed only in male and female gonads.. Hum Mol Genet.

[7] Elis S, Batellier F, Couty I, Balzergue S, Martin-Magniette ML (2008). Search for the genes involved in oocyte maturation and early embryo development in the hen.. BMC Genomics.

[8] Houston DW, Zhang J, Maines JZ, Wasserman SA, King ML (1998). A Xenopus DAZ-like gene encodes an RNA component of germ plasm and is a functional homologue of Drosophila boule.. Development.

[9] Johnson AD, Bachvarova RF, Drum M, Masi T (2001). Expression of axolotl DAZL RNA, a marker of germ plasm: widespread maternal RNA and onset of expression in germ cells approaching the gonad.. Dev Biol.

[10] Maegawa S, Yasuda K, Inoue K (1999). Maternal mRNA localization of zebrafish DAZ-like gene.. Mech Dev.

[11] Xu H, Li M, Gui J, Hong Y (2007). Cloning and expression of medaka dazl during embryogenesis and gametogenesis.. Gene Expr Patterns.

[12] Cheng MH, Maines JZ, Wasserman SA (1998). Biphasic subcellular localization of the DAZL-related protein boule in Drosophila spermatogenesis.. Dev Biol.

[13] Otori M, Karashima T, Yamamoto M (2006). The Caenorhabditis elegans homologue of deleted in azoospermia is involved in the sperm/oocyte switch.. Mol Biol Cell.

[14] Xu EY, Lee DF, Klebes A, Turek PJ, Kornberg TB (2003). Human BOULE gene rescues meiotic defects in infertile flies.. Hum Mol Genet.

[15] Karashima T, Sugimoto A, Yamamoto M (2000). Caenorhabditis elegans homologue of the human azoospermia factor DAZ is required for oogenesis but not for spermatogenesis.. Development.

[16] Maegawa S, Yamashita M, Yasuda K, Inoue K (2002). Zebrafish DAZ-like protein controls translation via the sequence ‘GUUC’.. Genes Cells.

[17] Peng JX, Xie JL, Zhou L, Hong YH, Gui JF (2009). Evolutionary conservation of Dazl genomic organization and its continuous and dynamic distribution throughout germline development in gynogenetic gibel carp.. J Exp Zool B Mol Dev Evol.

[18] Xu H, Li Z, Li M, Wang L, Hong Y (2009). Boule is present in fish and bisexually expressed in adult and embryonic germ cells of medaka.. PLoS One.

[19] Yamanoue Y, Miya M, Inoue JG, Matsuura K, Nishida M (2006). The mitochondrial genome of spotted green pufferfish Tetraodon nigroviridis (Teleostei: Tetraodontiformes) and divergence time estimation among model organisms in fishes.. Genes Genet Syst.

[20] Yoshizaki G, Sakatani S, Tominaga H, Takeuchi T (2000). Cloning and characterization of a vasa-like gene in rainbow trout and its expression in the germ cell lineage.. Mol Reprod Dev.

[21] Yoshizaki G, Takeuchi Y, Sakatani S, Takeuchi T (2000). Germ cell-specific expression of green fluorescent protein in transgenic rainbow trout under control of the rainbow trout vasa-like gene promoter.. Int J Dev Biol.

[22] Takeuchi Y, Yoshizaki G, Kobayashi T, Takeuchi T (2002). Mass isolation of primordial germ cells from transgenic rainbow trout carrying the green fluorescent protein gene driven by the vasa gene promoter.. Biol Reprod.

[23] Okutsu T, Shikina S, Kanno M, Takeuchi Y, Yoshizaki G (2007). Production of trout offspring from triploid salmon parents.. Science.

[24] Takeuchi Y, Yoshizaki G, Takeuchi T (2004). Biotechnology: surrogate broodstock produces salmonids.. Nature.

[25] Timmermans LP, Parmentier HK, van den Boogaart JG (1985). Surface markers of male germ cells in early development of carp (Cyprinus carpio L.) and the blood-testis barrier in fish. A study with monoclonal antibodies and horseradish peroxidase (HRP).. Cell Biol Int Rep.

[26] Liu WS, Wang A, Uno Y, Galitz D, Beattie CW (2007). Genomic structure and transcript variants of the bovine DAZL gene.. Cytogenet Genome Res.

[27] Kloc M, Etkin LD (2005). RNA localization mechanisms in oocytes.. J Cell Sci.

[28] Iwamatsu T (1988). Oogenesis in the Medaka Oryzias latipes -Stages of Oocyte Development.. Zoological Sci.

[29] Xu H, Li M, Gui J, Hong Y (2010). Fish germ cells.. Sci China Life Sci.

[30] Knaut H, Pelegri F, Bohmann K, Schwarz H, Nusslein-Volhard C (2000). Zebrafish vasa RNA but not its protein is a component of the germ plasm and segregates asymmetrically before germline specification.. J Cell Biol.

[31] Yoon C, Kawakami K, Hopkins N (1997). Zebrafish vasa homologue RNA is localized to the cleavage planes of 2- and 4-cell-stage embryos and is expressed in the primordial germ cells.. Development.

[32] Shinomiya A, Tanaka M, Kobayashi T, Nagahama Y, Hamaguchi S (2000). The vasa-like gene, olvas, identifies the migration path of primordial germ cells during embryonic body formation stage in the medaka, Oryzias latipes.. Dev Growth Differ.

[33] Xu H, Gui J, Hong Y (2005). Differential expression of vasa RNA and protein during spermatogenesis and oogenesis in the gibel carp (Carassius auratus gibelio), a bisexually and gynogenetically reproducing vertebrate.. Dev Dyn.

[34] Kito G, Aramaki S, Tanaka K, Soh T, Yamauchi N Temporal and Spatial Differential Expression of Chicken Germline-specific Proteins cDAZL, CDH and CVH During Gametogenesis.. J Reprod Dev.

[35] Ruggiu M, Speed R, Taggart M, McKay SJ, Kilanowski F (1997). The mouse Dazla gene encodes a cytoplasmic protein essential for gametogenesis.. Nature.

[36] Kee K, Angeles VT, Flores M, Nguyen HN, Reijo Pera RA (2009). Human DAZL, DAZ and BOULE genes modulate primordial germ-cell and haploid gamete formation.. Nature.

[37] Liu L, Hong N, Xu H, Li M, Yan Y (2009). Medaka dead end encodes a cytoplasmic protein and identifies embryonic and adult germ cells.. Gene Expr Patterns.

